# Cervical cancer prevention: Feasibility of self-sampling and HPV testing in rural and urban areas of Bolivia: An observational study

**DOI:** 10.1371/journal.pone.0292605

**Published:** 2024-03-07

**Authors:** Francesca Maria Carozzi, Ricardo Royder Yanez, Irene Paganini, Cristina Sani, Stefania Cannistrà, Marzia Matucci, Sandra von Borries, Silvia Traina

**Affiliations:** 1 Regional Laboratory of Cancer Prevention Unit, Institute for Cancer research, Prevention and Oncological Network ISPRO, Florence, Italy; 2 Italian Agency for Development Cooperation (AICS), La Paz Regional Site, Bolivia; University of Central Florida, UNITED STATES

## Abstract

**Background:**

Cervical cancer is a major health problem in Latin America. In 2019, the Italian Agency for Development Cooperation (La Paz regional site) conducted a pilot study to estimate the prevalence of high-risk human papillomavirus (HPV) and the feasibility of HPV screening in Bolivia through self-sampling and portable and transportable laboratory instruments for HPV testing in urban and rural areas.

**Methods:**

Women aged 20–65 years from La Paz (urban area), Toro Toro (rural area), and Acasio (rural area) were enrolled in local public health centers between Dec 1, 2019, and June 30, 2021. Self-sampling was carried out with the Viba-Brush system (Rovers, Oss, Netherlands) and samples were preserved in ThinPrep containers (Hologic Corporation, San Diego, CA, USA). The GeneXpert system (Cepheid, Sunnyvale, CA, USA) for high-risk HPV testing detects HPV E6 and E7 DNA via real-time PCR in a mobile system of easy execution requiring minimal manual intervention. The system provides results in about 1 h. The hr- HPV prevalence data, overall and partial genotyping, were analyzed considering the following age groups: 20–34, 35–44, and 45–65 years old.

**Findings:**

2168 women were enrolled: 614 (28.3%) in La Paz, 743 (34.3%) in Toro Toro, and 811 (37.4%) in Acasio. Only one sample was collected from each participant. 2043 (94.2%) of 2168 samples were adequate for HPV testing. 255 (12.5%) samples were positive for high-risk HPV. Comparing the urban area (La Paz) versus rural combined areas (Acasio+Toro Toro), using a logistic model, the HPV total rate was statistically significantly higher in the city of La Paz (15.0% vs 11.4%; OR:1.37;95% CI: 1.04–1.80). Furthermore, the HPV prevalence was declining by age, and the urban/rural odds ratio was 1.50; (95% IC 1.13–19). The overall HPV 16 positivity was 2.7% (55/2043) and for HPV 18/45 was 1.8% (37/2043) without any statistically significant differences between the three BHU enrolling centers. Only the prevalence of HPV group ‘39/56/66/68’ was significantly higher in La Paz (p<0,001) in comparison to Acasio and Toro Toro.

**Interpretation:**

The total and age-adjusted prevalence of high-risk HPV infection in rural and urban areas in Bolivia, as measured with a validated test for screening, is similar to that observed in Europe and the USA. Our study shows that a screening protocol for HPV testing with self-sampling would be feasible in urban and rural areas in Bolivia, and that the reported high occurrence of cervical cancer in Bolivia is not related to a higher rate of high-risk HPV infections. Carrying out HPV tests locally avoids the issues associated with transportation and storage of the collected material and allows the participant to wait in the clinic for the test result, overcoming the very long response time for screening test in Bolivia.

## Introduction

Cervical cancer ranks the fourth most common cancer in women with an estimated age-standardised incidence of cervical cancer of 13.1 per 100,000 women globally, variable widely among countries, with rates ranging from less than 2 to 75 per 100,000 women [1]. In 2018, in a greatly changing preventive landscape, the WHO launched an important call to all nations of the world for mobilising resources to make an end to suffering from cervical cancer [2] with the aim of reducing the risk of cervical cancer to less than 4 per 100,000 women worldwide using three approaches: vaccinating 90% of all girls by age 15 years, screening twice 70% of women ranging 35–45 years, and treating at least 90% of all precancerous lesions detected during screening.

Thanks to the resources provided by the WHO call to action, in the decades to come, the global scale-up of HPV vaccination and HPV-based screening—including self-sampling—could make cervical cancer a rare disease [1].

Bolivia shows one of the highest cervical cancer mortality rates in the world, with an estimated age-standardised incidence rate of about 36.6 cases per 100,000 persons/year and an age-standardised mortality rate of 18 cases per 100,000 persons [3], indeed about 1,985 new cervical cancer cases are diagnosed annually (estimates for 2020) and Cervical cancer ranks as the 1^st^ leading cause of female cancer [3]. To face the high number of cervical cancer in Bolivian women, from 2017, the Bolivian Ministry of Health promoted a national vaccination program against human Papilloma virus (HPV), targeting girls aged 10–12 years, that reached approximately 64% coverage [4] and a screening program with Pap test every three years showing about 33% coverage [5]. However, cytology-based screening programs have been less successful in Low and Middle Income Countries (LMIC), because of the complex logistical, technological, and human resource requirements, with the need of new approaches to overcome the obstacles and obtain an effective screening. The knowledge that persistent infection with carcinogenic human papillomavirus (HPV) types is the main cause in triggering the development of cervical cancer has opened new pathways for primary and secondary prevention [6]. In particular, the World Health Organization has recommended molecular testing searching for human papillomavirus (HPV) for primary screening of cervical cancer in LMIC [7] which show better sensitivity than cytology or visual inspection with acetic acid, for the detection of cervical neoplasia [8, 9].

The other important obstacle to the Cervical Cancer screening in LMIC concerns the sample collection. Indeed, the standard method of obtaining samples for HPV testing is by a clinician who collects a cervical sample during a gynaecological examination but women might be reluctant to go for screening or might live in areas hard to reach. The self-sampling offers the opportunity to overcome these issues and, at the same time, the shortages of staff in health centres across LMIC [10, 11]. Indeed, many studies have already shown that self-sampling shows a high acceptance by women in many different settings [12–15].

Finally, wide variations in the prevalence of high-risk (HR) human Papillomavirus (HPV) and in the specificity of HPV DNA tests for cervical precancerous lesions have been reported previously [16].

However, while several studies on HPV prevalence in South American Countries [17] have been already published, only limited data were available for HPV prevalence in Bolivian women [18–20].

In 2019, the Italian Agency for Development Cooperation in La Paz (AICS, a government agency under the Italian Ministry of Foreign Affairs and International Cooperation), in partnership with the Ministry of Health (MOH) of Bolivia, implemented a pilot study in order to assess feasibility, outcomes and challenges of HPV-based cervical cancer screening on self-collected samples among women living in rural and urban areas of the country. In this paper we report the prevalence of hr HPV infection, analysed with a validated test for screening purpose, in women aged 20–65 years.

## Materials and methods

### Study population and recruitment

The pilot project on HPV screening by self-sampling (observational study) enrolled women aged 20–65 years in Centro de Salud “El Rosal”, Zona Llojeta del Municipio de La Paz (urban area), Centro de Salud in Toro Toro (rural area) and Centro de Salud in Acasio (rural area). The standard population eligible for screening is women aged 25 to 64. However, considering the early starting of Bolivian women to sexual activity and the high incidence of cervical cancer in younger women in Bolivia (15–44 years old) (reference (ICO/IARC Information Centre on HPV and Cancer (HPV Information Centre Human Papillomavirus and Related Diseases in the World. Summary Report 10 March 2023) the screening eligible population was settled for the 20–64 age group.

The population of the involved areas have been informed about the opportunities of the HPV-based cervical cancer screening, using radio messages, flyers and billboards. Women that fulfilled the age criteria, decided voluntarily and spontaneously to participate in the HPV screening project without receiving a direct invitation letter or a personal call. Moreover, local Health Workers (HWs) of the 2 enrolled Basic Health Units (BHU) of Acasio and Toro Toro played a more active role, realising a “door to door” campaign in the more isolated and difficult to reach villages surrounding the health centres. All women attending the three local public health centres between Dec 1, 2019, and June 30, 2021 were invited by HWs to participate in the pilot project for cervical cancer prevention screening using high risk HPV test on self-collected samples.

In particular, sexually active women between the ages of 20 and 65 who were not pregnant, not hysteroctomized, and not treated for cervical lesions were eligible and, after giving their verbal informed consent to participate in the pilot program and share their results with the medical staff, received the self-test device.

The HWs were trained to be able to provide additional information about the pilot study and self-sampling procedure in order to answer all women’s doubts before starting the collection. After collection, HWs in BHUs performed the HPV test, printed the result and gave it to the medical staff in the BHUs. The clinicians shared the results with women and, in case of a positive test, invited women to perform a Pap Test or a colposcopy, depending on the HPV type detected.

The pilot project was defined by AICS in collaboration with the Bolivian Ministry of Health and approved by both parties through the signing of an agreement.

**Fig 1:**
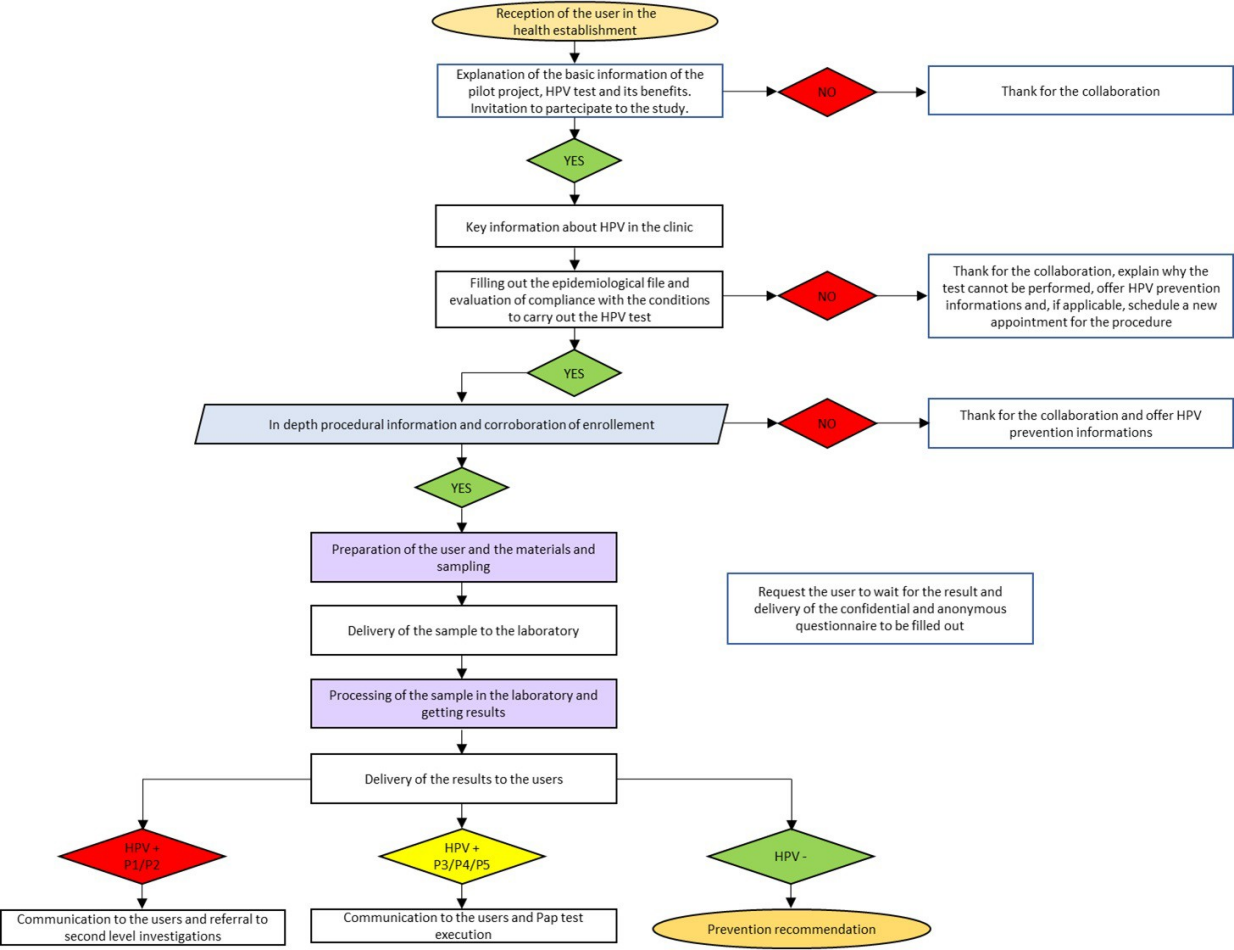
Flow chart of AICS-Pilot HPV project.

The flow-chart of the project is shown in Fig 1.

### Ethical aspects

The Ministry of Health in Bolivia approved the protocol and declared its compliance to Bolivian ethical principles (MSyD/VGSS/DGRSS/CE/145/2022). Moreover, the pilot project was considered as minimal risk of adverse complications given that the self-sampling procedures, using a standard practice in cervical cancer screening programmes, is minimally invasive and associated with very low discomfort for women. All the women enrolled were informed about the aims of the pilot project and provided a verbal informed consent to participate in HPV screening for cervical cancer prevention using self-sampling and to share test results with medical staff. Women that agreed to participate in the HPV program for cervical cancer screening received the device to perform the vaginal self-sampling.

### Sample collection

Women performed the self-collection directly in BUHs, in a private clinical examination room using a Viba-Brush® (Rovers, Oss, Netherlands), after receiving detailed instructions by a community HW. The self-collected sample was collected inserting and rotating the tip swab into the vagina and then placing it in a Thinprep® vials (Hologic Corporation, San Diego, CA, USA). After collection, samples were stored in BHUs at room temperature, according to manufacter’s instruction, until HPV test was performed; then they were discarded.

### HPV test

The HPV test was directly performed in each local BHUs using GeneXpert HPV HR (Cepheid, Sunnyvale, California, USA), a HPV test validated for screening purposes, with a sensitivity for CIN2+ of 98.7% and a specificity of 82.3% [21]. The Genexpert HPV HR system allows the detection of the E6/E7 genes of 14 HPV types by Real Time PCR, in about 1 hour, using closed cartridges with a minimal manual intervention by the operator. In case of positive results to hr-HPV, the GeneXpert HPV HR produces a partial genotyping result, distinguishing HPV 16 positive samples from those positive for other types, divided into four mixed groups HPV 18/45, HPV 31/33/35/52/58, HPV 51/59 and HPV 39/56/66/68.

Moreover, an internal quality control of sample adequacy is provided.

Each BHU has been equipped with a small portable GeneXpert instrument able to carry out among 1 to 4 tests simultaneously.

The advantages of this test are that to use the GeneXpert instrument there is no need for specialised skills as well as the speed and accuracy in obtaining the results (one hour) make it an appropriate test in a screen-and-treat setting. The operational stage only consists of putting 1.5ml of the self-cervical material into Xpert HPV cartridge, which is then slotted into the GeneXpert machine and pushing the “start button”. The HWs were trained properly for the use of this HPV molecular test equipment and for printing the results, according to the manufacturer’s instructions.

GeneXpert HPV is a World Health Organization prequalified test and has European Conformity Marking (CE marking). It is available globally and is registered for use in many countries. GeneXpert HPV is included in the 2020 list of human papillomavirus assays suitable for primary cervical cancer screening [21].

### Data collection and statistical analyses

Hr HPV results were analysed considering the positivity to any HPV type or to the 5 HPV types groups provided by GeneXpert HPV HR test separately, by classes of age and by the three enrolled centres. The following age groups were considered: 20–34, 35–44 and 45+. The choice of using HPV Test as a screening test for all the screening population is based on the very low coverage (20–35%) of the screening using Pap test in Bolivia; notwithstanding, the differences in screening population were taken into account for age grouping. Indeed the 20–34 age class represents women screened in Europe using Pap smear due to the high frequency of HPV positive tests. The 45+ age class included women with the lower frequency of HPV positivity [4], while 35–44 age class represents the intermediate group. Women with missing age were not included in the age-positive assessments for HPV group.

If a sample resulted positive for more than one of the HPV types groups identifiable by the GeneXpert HPV HR test, it was considered as co-infection. A self-collected sample was defined valid if one or more HPV channels gave a positive result or if internal quality control for human gene (beta globin) resulted positive. All data were imported and analysed in STATA version 12 (StataCorp. Stata Statistical Software: Release 12. College Station, TX: StataCorp LP.2011.).

The overall HPV prevalence statistical analysis and HPV prevalence by genotyping was performed using the *proportion* STATA command to estimate 95% Intervals of Confidence. The STATA logistic analysis was carried out by BHU or grouped by urban (La Paz) and rural (Acasio and Toro Toro), adjusted by age (continuous) or by age groups (<35,35–44, 45+). All p-value less than 0.05 was considered statistically significant.

## Results

The pilot project enrolled 2168 women, of whom 811 (37.4%) in Acasio, 614 (28.3%) in La Paz and 743 (34.3%) in Toro Toro.

HPV test showed a valid result for 2043 (94.2%) samples, while 125 (5.8%) samples resulted invalid (Table 1) with an higher number of invalid results in Toro Toro (13.6%) than in Acasio (1,97%) and La Paz (1.3%).

**Table 1:** Valid and invalid HPV test by centre and overall.

BHU	Valid HPV results	Invalid HPV results	Total
**ACASIO**	795 (98.0%)	16 (1.97%)	811
**LA PAZ**	606 (98.7%)	8 (1.3%).	614
**TORO TORO**	642 (86.4%)	101 (13.6%)	743
**TOTAL**	2043 (94.2%)	125 (5.8%)	2168

Number of valid HPV samples, percentage (%) shown within each enrolling centers

Table 2 shows the number of women enrolled, with a valid HPV test, by age group in the three enrolled BHUs.

**Table 2:** Enrolled women by centre (BHU) and age group.

BHU	Age group n. (%)			
	20–34	35–44	45+	Missing age
**ACASIO**	333 (41.9%)	207 (26.0%)	182 (22.9%)	73 (9.2%)
**LA PAZ**	274 (45.2%)	185 (30.5%)	147 (24.3%)	0 (0.0%)
**TORO TORO**	331 (51.6%)	179 (27.9%)	115(17.9%)	17 (2.7%)
**TOTAL**	938 (45.9%)	571 (27.9%)	444(21.7%)	90 (4.4%)

Pearson chi2 (6) = 88.7692 Pr = 0.000 Number of participants with a valid HPV test, by age group, percentage (%) shown within each enrolling Health Unit (BHU)

The mean age of enrolled women is similar in La Paz (mean: 37.0; SD:9.9) and Acasio (mean: 36.5; SD:10.7), while the mean age in Toro Toro was lower (mean:34.2; SD:9.7). However, the date of birth has not been recorded for about 10% of enrolled women in Acasio, while in Toro Toro this percentage decreases to 2.6% and no missing ages were reported in La Paz.

The overall hr-HPV positivity rate, showed in Table 3, was 12.5% (255/2043) but dividing by single BHU, a lower HPV positivity is observed in the two rural areas of Acasio 10.6% (84/795) and Toro Toro 12.5% (80/642), compared to women enrolled in La Paz 15.0% (91/606). When comparing the urban area (La Paz), versus the combined rural areas (Acasio + Toro Toro), the total HPV rate was significantly higher in the city of La Paz (15.0% vs 11.4%) and the logistic analysis performed showed a significantly higher overall HPV positivity rate in the urban (La Paz) versus rural BHUs [OR = 1.37; 95% IC (1.04–1.80)].

**Table 3:** HPV HR prevalence by BHU.

BHU	HR-HPV NEG	HR-HPV POS	N. Total
	n. (%)	n. (%)	
		95%CI	
**ACASIO**	711 (89.4%)	84 (10.6%)	795
		95%CI: 8.4%-12.7%	
**LA PAZ**	515 (85.0%)	91 (15.0%)	606
		95%CI: 12.2%-17.9%	
**TORO TORO**	562 (87.5%)	80 (12.5%)	642
		95%CI: 9.9%-15.0%	
**TOTAL**	1788 (87,5%)	255 (12.5%)	2043
		95%CI: 11.1%-13.9%	

Chi-square (2) = 6,2; p = 0.04

Number of HPV positive and HPV negative results, prevalence (%) and 95% confidence intervals shown within each enrolling Health Unit (BHU)

Women with no registered age were excluded by the statistical analysis of HPV positivity by age; however, as expected on the basis of HPV epidemiology in other countries, the observed prevalence was higher in younger women and decreasing by age, from 15.5% in 20–34 age group to 8.2% in the 45+ age group and, adjusting by age, the risk ratio urban vs rural was 1.50 (95% CI: 1.13–1.99), confirming the difference between the rural and urban residence women. HPV prevalence by age groups and centres are shown in Table 4.

**Table 4:** HR HPV positivity (%) by age and BHU.

BHU	20–34	35–44	45+	Total	p-value
**ACASIO**	46/333 (13.8%)	14/207 (6.8%)	15/182 (8.2%)	75/722 (10.4%)	<0.05
**LA PAZ**	50/274 (18.3%)	25/185 (13.5%)	16/147 (10.9%)	91/606 (15.0%)	<0.05
**TORO TORO**	49/331 (14.8%)	21/179 (11.7%)	6/115 (5.2%)	76/625 (12.2%)	<0.05
**ALL**	145/938 (15.5%)	60/571 (10.5%)	37/444 (8.3%)	242/1953 (12.4%)	<0.05

Number of HPV positive women by age, prevalence (%) shown within each BHU

The overall HPV 16 positivity was 2.7% (55/2043) and for HPV 18/45 was 1.8% (37/2043) without any statistically significant difference between the three BHUs enrolling centres. Only the prevalence of HPV group ‘39/56/66/68’ was statistically significant higher in La Paz (p<0,05) compared to Acasio and Toro Toro (Table 5). Moreover, HPV partially genotyping showed a trend by age for all HPV groups, with statistically significant differences for HPV 18/45, HPV 31/33/35/52/58 and HPV 51/59 (Table 6).

**Table 5:** Frequency of partial HPV genotyping by BHU and overall.

BHU	HPV 16	HPV 18/45	HPV 31/33/35/52/58	HPV 51/59	HPV 39/56/66/68
	n. (%)	n. (%)	n. (%)	n. (%)	n. (%)
**ACASIO**	22/795 (2.8%)	12/795 (1.5%)	39/795 (4.9%)	19/795 (2.4%)	13/795 (1.6%)
	95%CI: 1.6%-3.9%	95%CI: 0.7%-2.4%	95%CI: 3.4%-6.4%	95%CI: 1.3%-3.5%	95%CI: 0.8%-2.5%
**LA PAZ**	13/606 (2.2%)	16/606 (2.6%)	30/606 (4.9%)	19/606 (3.1%)	29/606 (4.8%)
	95%CI: 0.99%-3.3%	95%CI: 1.4%-3.9%	95%CI: 3.2%-6.7%	95%CI: 1.7%-4.5%	95%CI: 3.1%-6.5%
**TORO TORO**	20/642 (3.1%)	9/642 (1.4%)	38/642 (5.9%)	18/642 (2.8%)	13/642 (2.0%)
	95%CI: 1.8%-4.5%	95%CI: 0.5%-2.3%	95%CI: 4.1%-7.8%	95%CI: 1.5%-4.1%	95%CI: 0.9%-3.1%
**TOTAL**	55/2043 (2.7%)	37/2043 (1.8%)	107/2043 (5.2%)	56/2043 (2.7%)	55/2043 (2.7%)
	(95%CI:2%-3.4%)	95% CI:1.2%-2.4%	(95%CI:4.3% -6.2%)	(95%CI:2.0%-3.4%)	(95%CI:2%-3.4%)
**p-VALUE**	0.56	0.19	0.64	0.69	<0.05

Partial genotyping results by BHU and overall: number of positive women, percentage % and 95% confidence intervals

**Table 6:** Genotyping results by age (all BHU).

	20–34	35–44	45+	TOTAL	p-value
**HPV 16**	27 (2.9%)	17 (3.0%)	7 (1.6%)	51	0.29
**HPV 18/45**	26 (2.8%)	3 (0.5%)	6 (1.4%)	35	<0.05
**HPV 31/33/35/52/58**	66 (7.0%)	21 (3.7%)	16 (3.6%)	103	<0.05
**HPV 51/59**	34 (3.6%)	12 (2.1%)	4 (0.9%)	50	<0.05
**HPV39/56/66/68**	31 (3.3%)	13 (2.3%)	6 (1.4%)	50	0.08
**TOTAL**	938	571	444	1953	

Partial genotyping results by age groups in the three BHUs and overall: number of positive women and percentage (%)

The Table 6 shows the HPV partially genotyping by age.

The results showed an evident trend by age for all HPV groups, with statically significant differences for HPV 18/45, HPV 31/33/35/52/58 and HPV 51/59.

**Fig 2:**
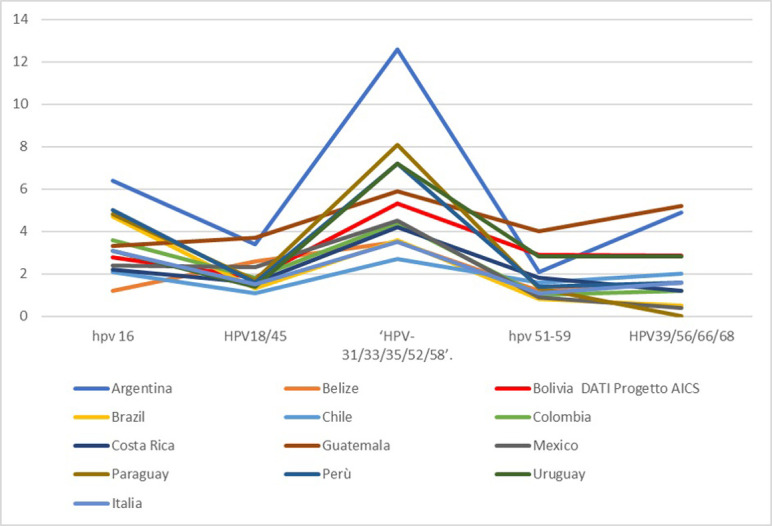
Comparison of HPV type distribution Bolivia vs South American countries and Italy (Bolivia AICS project data, other countries ICO_HPV CENTER data updated 2021) according to partial genotyping allowed by HPV method applied in AICS_HPV_Bolivia_Project.

Finally, in Table 7 we compare HPV partial genotyping data obtained in this project with other Latin American countries referring to data from table n.22 of ICO_HPV Center Report [4]; these data has been also graphically represented in Fig 2. Considering that the HPV Test used in this pilot project allows only partial HPV genotyping by subdividing the 14 HPV types into 5 groups, we grouped data of the single HPV genotype reported for each country into the 5 HPV types groups of GeneXpert, to allow a better comparison between the HPV genotyping data results. The prevalence of HPV 16 in Latin America shows some variability ranging from 1.2% in Belize to 6.4% in Argentina, with Bolivia showing an intermediate value of 2.7%. The frequency of HPV 18/45 group is about a half of HPV 16, except for Guatemala where HPV 18/45 shows a higher frequency than HPV 16. The group including HPV 31/33/35/52/58 shows a higher prevalence in all countries except for Brazil, where HPV 16 is the most frequent type overall. In Bolivia, Guatemala and Costa Rica a higher frequency of HPV 51–59 is observed than in the other countries. Finally, a higher frequency of HPV 39/56/66/68 is observed in Bolivia, Guatemala and Argentina.

**Table 7:** Type-specific prevalence in Latin America and Italy and comparison with HPV_AICS_Bolivia pilot project data. Genotyping data from Latin American nations and Italy were extracted from ICO _AIRC 2021 table n. 22 and they were grouped into the 5 HPV groups (P1-P2-P3-P4-P5) identified with the HPV test used in the AICS_Bolivia project.

Country (n. people)	HPV 16	HPV 18/45	HPV 31/33/35/52/58	HPV 51/59	HPV 39/56/66/68*
**Bolivia AICS_PILOT STUDY (n. 2043)**	**2.7**	**1.8**	**5.2**	**2.7**	**2.7**
**Belize (n. 426)**	1.2	2.6	3.5	1.2	1.6
**Argentina (n. 1908)**	6.4	3.4	12.6	2.1	4.9
**Brazil (n.1364)**	4.7	1.3	3.6	0.8	0.5
**Chile (n.913)**	2.1	1.1	2.7	1.6	2.0
**Colombia (n.2138)**	3.6	1.8	4.4	1.0	1,0
**Costa Rica (n. 7459)**	2.2	1.6	4.2	1.8	1.2
**Guatemala (n. 274)**	3.3	3.7	5.9	4.0	5.2
**Mexico (n. 8089)**	2.4	2.3	4.5	0.9	0.4
**Paraguay (n. 293)**	4.8	1.7	8.1	1.3	0
**Perù (n. 879)**	5.0	1.6	7.2	1.4	1.6
**Uruguay (n. 1119)**	3.1	1.4	7.2	2.8	2.8

*HPV 66 and HPV 68 are not present in ICO _AIRC

## Discussion

Cervical cancer represents the main cause of death of women in Bolivia [3]. However, compared to other Latin American countries, data on HPV prevalence are scarce and fragmented and often not representative of the great socio-environmental variability of the Bolivian territory [18–20]. Therefore, the Bolivian Ministry of Health promoted a national vaccination program against Human Papilloma Virus (HPV), and a screening program with Pap test every three years; but while the vaccination reached almost the 64% of the selected population [4], screening obtained only about 33% coverage [5], probably because of the poor success of cytology-based screening programs in Low and Middle Income Countries.

This pilot study, promoted by AICS and the Bolivian Ministry of Health, aims to evaluate the feasibility of a screening protocol that includes sample collection with self-sampling, to address socio-cultural barriers, and an easy-to-use molecular HPV test exploitable also in local health centres, to obtain representative HPV infection prevalence data of the Bolivian territory.

In this paper we reported the HPV prevalence obtained in over 2000 women (age 20–65 years old) living in 3 different areas of Bolivia: Toro Toro (rural area), Acasio (rural area) and in La Paz (urban area). The data were collected in a pilot study conducted in 2019 from the Italian Agency for Development Cooperation (La Paz regional site), in agreement with Bolivian Ministry of Health, with the aim of estimating the feasibility of HPV screening in Bolivia through self-sampling and portable and transportable laboratory instruments for HPV testing in urban and rural areas. This study started at the end of 2019, so the SARS-CoV-2 pandemic in 2020 and 2021 inevitably had a negative impact on the enrolment. However, this pilot study represents, to the best of our knowledge, the largest study carried out in Bolivia reporting the assessment of the prevalence of HPV infections. In our pilot project the prevalence of high-risk HPV, assessed by a validated HPV test for screening, was 12.5% with a seeming statistically significant difference between rural and urban areas [OR = 1.37; 95% IC (1.04–1.80)]; nevertheless, considering the similar absolute values of the HPV positivity percentages (15.0% vs 1.4%) we cannot exclude that this difference could be actually attributable to the unlike number of cases in the two groups (1437 in rural areas vs 606 in La Paz).

The assessed HPV prevalence in Bolivia is similar to what described in other studies performed in Latin American countries using self-sampling and HPV test validated for screening: Mexico, 11.6% [22], Brasil, 11.5% [23] and Guatemala, 12.4% [24] but lower than what has been reported in Argentina, 15.2% [25], Honduras, 14.5% [24], Nicaragua, 14.2% [24] and Colombia, 21.3% [26]. Otherwise, data from Europe and other developed countries show a more variable rate of overall HPV positivity on self-sampling ranging from 8.2% in the Netherlands [27] and 9.0% in Australia [28] to 12.4% in Italy [29] or 13.0% in Denmark [30]. Finally, a recent meta-analysis on self-sampling reported that the overall positivity rate to HPV in self-sampling studies around the world is 11.1% [10]. Furthermore, as expected [4], HPV prevalence decreases by age from 15.5% in the 20–34 age group to 8.2% in the 45+ age group.

Current HPV tests for screening include 13–14 HPV genotypes that vary substantially in their association with cervical cancer and precancerous lesions. In cervical cancer, by far, the most important type is HPV 16, followed by HPV 18 [31]. However, several studies showed that genotypes 31, 33, 52, and 58 confer risks similar to HPV 18 and 45, thereby establishing impetus for contemplating more complex screening algorithms using genotype-specific risk stratification to allow for more precise colposcopy referral recommendations and reducing over treatment [32–37].

Recently, an alternative approach suggests to adopt a system of HPV clusters (with low, medium and high oncogenic potential) defining discrete patient risk groups with increased precision: HPV 16, 31, 33, HPV 18, 52, 35, 58 and HPV 51, 68, 45, 39, 66, 56, 59 were articulated as having high, medium and low potential for pre-invasive high grade lesions respectively [38].

These insights in screening protocol induced adjustments in technological development increasing the number of automated assays that offer type-specific read-outs beyond 16/18.

The HPV test used in this project falls into this type of new tests. Genexpert HPV HR is a test validated for screening purpose and able to perform immediately a partial genotyping, subdividing the 14 HR HPV types in five groups: group 1 HPV 16, group 2 HPV 18 / 45, group 3 HPV 31/33/35/52/58, group 4 HPV 51/59 and group 5 HPV 39/56/66/68.

In our study, the group which comprises HPV-31/33/35/52/58 types showed the highest frequency in all enrolment centres, while in literature, only 3 small studies assessed the prevalence of type-specific HPV in Bolivia. In 2001, Lema and co-workers [18] found an HPV prevalence of 8.0% in 151 enrolled women and, genotyping results conducted by RLFP method, showed that the most frequent types were HPV 31 and HPV 58. In 2014, Terán Calderón [19] enrolled 868 women reporting a prevalence of 18.1% with HPV 16 as the most prevalent type (3.7%), followed by 31, 52 and 51. In 2020, Patzi-Churqui and co-workers [20] enrolled 364 women and reported a prevalence by genotype very similar to those of our study, even taking into account the different method used for testing: the most prevalent high-risk HPV types were HPV 56, 39 and 31, followed by HPV 16 and 18. The genotyping results of our survey are consistent with data reported in these studies.

Moreover, we compared the genotyping results in 5 groups obtained in this pilot project with genotyping data from other Latin American countries. To allow a better comparison between the genotyping results obtained in this pilot project with data from other Latin American countries, USA and Europe we used the data from table 22 in ICO HPV centre report [4] grouping data of the single HPV genotypes reported for each country into the 5 classes of HPV type used by GeneXpert. The comparison confirms that the prevalence of the main HPV genotypes in Bolivia is similar to that obtained in other Latin American countries. Notably, the high prevalence of HPV 31/33/35/52/58 reported in our study highlights the importance of implementing vaccination with the new nonavalent vaccine, even if vaccines of first generation (bivalent and quadrivalent) showed common cross hybridization for types 31, 33, 35.

Finally, the number of invalid samples in this study was higher (5.8%) than how reported in literature (0.7%) [19], despite the high sensitivity of the HPV test used (98.7%). However, the high number of invalids can only be attributed to the Toro Toro centre, which reported 13.6% of invalid samples, while the other two centres showed about 1.5%. Moreover, the majority of the invalid results reported in Toro-Toro BHU were obtained during the first three months of the project. Deepening the causes of this higher rate in Toro Toro, it was highlighted that while inadequate samples were very low 1.1% (23/2043), the most of invalid results were technical inadequate (4.5%) (message of error: “error result”) probably related to sample handling, feeding, cartridge/module, temperature and other errors not otherwise specified, as reported in manufacturer instruction. Based on these observations, even if the protocol had included a double sampling of cervical material from the women, a recovery in terms of valid results would not have been obtained. Additionally, during the implementation of the project, the performance of the HPV tests in Toro Toro improved, although the number of invalid results remained higher compared to the other two centres. These remarks suggest that the increase in the number of invalid tests is probably attributable to less experienced HWs in Toro Toro despite the ease of use of the laboratory instrumentation, and that BHU staff will need continued training and more support from experienced personnel.

### Strengths and weakness

This project started in December 2019 and it was practically carried out during the first SARS-CoV-2 pandemic period. This surely slowed down enrolment and made access to health centres more difficult especially for the in-depth examinations provided for HPV-positive women in the screening protocol. However, more than 2000 women were enrolled, representing the largest HPV prevalence study with partial genotyping carried out in Bolivia.

Another important aspect of this project is the choice of BHU in both rural and urban areas which made the study more representative of the entire Bolivian territory. Still, the difficulties in involving a more reluctant population and the lack of structured internal organisation of health facilities in rural areas, determined the missing of some important data like ages of participating women, and the number of invalid results due to lack of experience in molecular analysis of HWs.

On the other hand, the choice to perform the project in local Bolivian health centres may have reduced the adherence to the study compared to mailing the self-sampling kit directly to the women at home. However, door-to-door and radio information has been carried out before and during the start of the project, in order to inform the population as much as possible. Moreover, performing both the collection and the HPV test, at BHU, allowed women to get instructions directly from the health staff at the Centre on how to use the self-sampling kit and to receive, immediately (within 1 hour) the response of the screening test performed. This last approach definitely represents an added value for women participation because usually the Pap test, as a screening test, shows in Bolivia a very long time response, calculated in months.

The option of an HPV test simple to perform and not requiring special skills, allowed to execute the test directly in the health centre, certainly avoiding the issues associated with transportation and storage of samples.

Yet, the execution of the test by non-laboratory personnel caused a higher number of invalid samples than expected, especially in one of the rural centres. In fact, even though all the health centre staff had been trained in the same way for the test execution, machine operation and process error resolution, some operators had more difficulties causing a higher number of invalid results than others. The relatively higher number of inadequate tests detected suggests the need for ongoing training of health care workers involved in molecular testing and the importance of "widespread" monitoring of results.

The chosen HPV test, in addition to the overall HR HPV positivity data, provides partial genotyping, allowing to accurately assess the prevalence of HPV 16 and HPV 18/45. This important data will permit to verify the effectiveness of bi/quadrivalent HPV vaccination in Bolivia for preventing these infections, even if the assessment of the exact prevalence for the other high-risk genotypes was not allowed.

## Conclusions

In Bolivia, the prevalence of hr-HPV infection in vaginal self-sampling was very similar to the prevalence of HPV reported in European and developed countries and highlights the feasibility of a screening protocol based on HPV testing in self-collected samples in the country. This pilot project demonstrates the feasibility of performing HPV tests even in local health centres, using a fully automated real-time molecular biology system with minimal operator intervention and capable of providing an hr-HPV result within 60 minutes. This approach, especially suitable for low-income countries, also ensures that women can wait at the clinic or outpatient clinic to receive the screening test result immediately.

## Supporting information

S1 File (DOCX)

## Abbreviations

HPV: (Human papilloma Virus)
Hr-HPV: (High risk Human papilloma Virus)
BHUs: (Basic Health Units)
HWs: (health workers)
PCR: (polymerase chain reaction)
ISPRO: (Institute for cancer research, prevention and oncological network)–Florence (Italy)
AICS: (Italian Agency for Development Cooperation)
