# Supplementary material for: Cervical cancer prevention: Feasibility of self-sampling and HPV testing in rural and urban areas of Bolivia: An observational study
Source: PLoS One. 2024 Mar 7;19(3):e0292605. doi: 10.1371/journal.pone.0292605 (PMC10919649; doi:10.1371/journal.pone.0292605)
Supplement: S1 File — (DOCX) [file pone.0292605.s001.docx]

#### **Abbreviations:**

[HPV](https://www.thelancet.com/action/doSearch?AllField=%22HPV%22&ISSN=2667-193X) ([Human papilloma Virus](https://www.thelancet.com/action/doSearch?AllField=%22Human%20papillomavirus%22&ISSN=2667-193X))

Hr-HPV (High risk Human papilloma Virus)

BHUs (Basic Health Units)

Hws (health workers)

PCR (polymerase chain reaction)

ISPRO (Institute for cancer research, prevention and oncological network) – Florence (Italy)

AICS (Italian Agency for Development Cooperation)

**Keywords**

- HPV self-sampling
- Cervical cancer prevention
- Bolivia
- HPV prevalance
- Screening
